# Is It Harassment? Perceptions of Sexual Harassment Among Lawyers and Undergraduate Students

**DOI:** 10.3389/fpsyg.2020.01793

**Published:** 2020-08-21

**Authors:** Mally Shechory-Bitton, Liza Zvi

**Affiliations:** Department of Criminology, Ariel University, Ariel, Israel

**Keywords:** sexual harassment, lawyers, judgments, victim blame, just world theory

## Abstract

This study examined differences between lawyers (*n* = 91) and undergraduate students (*n* = 120) regarding their evaluation of behavior as sexual harassment (SH) and blame attributions toward offender and victim. The current study used a cross-sectional, comparative, independent measures design. Also examined was the correlation between these perceptions and belief in a just world (BJW) hypothesis. The respondents were presented with case descriptions of SH that were identical in all aspects but the perpetrator and victim’s gender (alternately depicted as male/female and female/male). Results showed that both lawyers and students agreed that the described event comprised SH, yet gender bias was evident. Both lawyers and students were more inclined to regard the behavior as SH when the vignette description depicted the perpetrator as a man (i.e., female victim) than as a woman (male victim). Gender bias was also evident in the examination of blame attributions, which were higher toward a male (vs. female) harasser. Nonetheless, the findings indicate that lawyers were less biased than students, manifested in less victim-blame and higher perpetrator blame attributions. No correlation between BJW and perceiving the vignette as SH and blame attribution was found. The findings indicate discriminatory judgments of SH based on gender. Gender-related stereotypes and sociocultural explanations are discussed.

## Introduction

The attitude of society in general, and of the law enforcement system in particular, is strongly linked to how victims cope with assaults perpetrated against them (Venema, 2016; Shaw et al., 2017; Debowska et al., 2018a; Boduszek et al., 2019a; Craig et al., 2020), and this is particularly true of the psychological and physiological consequences suffered by victims of sex offenses (Willness et al., 2007; Dworkin et al., 2017; Boduszek et al., 2019b). Many victims refrain from reporting the assault against them to the legal authorities due to the fear of not being believed and the fear of the stigma often attached to sex crime victims, which add to feelings of shame, embarrassment, degradation, guilt, and self-blame (Perilloux et al., 2014; Landström et al., 2016). Under-reporting is even more prevalent when the victims are men (Javaid, 2018). Due to embarrassment, concern of encountering distrust and concern of blame attribution, many men avoid reporting sexual offenses perpetrated against them, both slight and serious, to the authorities (Turchik and Edwards, 2012; Hammond et al., 2017).

Recognition of factors that might bias human perception has crucial significance in the forensic domain with regard to interrogating witnesses and suspects and reaching legal decisions (Kassin et al., 2013; Peer and Gamliel, 2013; Shechory-Bitton and Zvi, 2015, 2016). This is extremely significant in cases of sexual abuse, where the legal system must often decide between the version of the victim and that of the perpetrator (Shechory-Bitton and Jaeger, 2019). The negligible research that has explored this topic in law enforcement and the justice system focused mainly on police officer perceptions of rape incidents (see, for example, Sleath and Bull, 2012, 2015; Shechory-Bitton and Jaeger, 2019). The findings show that while neutrality and objectivity are stressed in these domains, real-life circumstances often suggest a different more biased reality. There is strong evidence that social attitudes affect conduct in the legal and justice systems, infecting them with myths and stereotypes common in general society (Rumney, 2009; Page, 2010; Willmott et al., 2018; Shechory-Bitton and Jaeger, 2019). For example, Temkin and Krahe (2007) conducted a detailed analysis on the impact of social attitudes on law enforcement in cases of rape perpetrated against women. Their research showed how the treatment of rape incidents by the criminal justice system is based on and influenced by stereotypical rape beliefs. This was found to be especially the case when strong evidence was lacking, allowing stereotypes and attitudes held by judges to register a pronounced impact (Lovett and Kelly, 2009).

As noted, research on the perceptions of sex offenses by legal professionals who work in the criminal justice sector is scarce. The objective of the current study was to add to the limited knowledge in this area by examining their perceptions toward sexual harassment (SH). While some countries refer to SH as purely a civil cause of action, other countries refer to it as a sex-based criminal offense. The concept of SH is ambiguous, with no clear consensus on a universally accepted definition (for review, see O’Leary-Kelly et al., 2009; Magaji et al., 2019). Many countries have enacted laws that prohibit SH. However, most are general and open to interpretation in the justice system. Legal definitions of SH vary, as countries have evolved their own legal definitions based on need and circumstance (Suma et al., 2017). In Israel, where this study was conducted, all behaviors of SH constitute a violation of criminal law. Hence, all cases of SH are tried in criminal court. In addition to the violation of criminal law, SH behaviors may also constitute a civil wrong, and the victim may sue the perpetrator in civil court for monetary damages. The purpose of the Israeli law is to prohibit SH in order to protect a person’s dignity, liberty, and privacy and to promote gender equality. The law addresses six situations that constitute SH: (1) extortion (using threats to coerce an individual into sexual activity), (2) indecent assault, (3) recurring sexual offers (multiple attempts by the harasser even after their original sexual proposition was rejected), (4) repeated references to a person focusing on sexuality (continuing even after the harasser was told to desist from said references), (5) disgraceful or degrading treatment of a person in relation to their sex or sexual orientation, and (6) posting a photograph, movie, or recording of a person, focusing on their sexuality, in circumstances in which the publication might humiliate or degrade the person, and without receiving consent for its publication (Prevention of Sexual Harassment Law 5758, 1998). As can be seen from the wording of the law, there may be quite a few situations where legal professionals working within the judicial system could find it difficult to decide whether or not SH has occurred.

Social perception of the concept varies as well. Despite legislation and increasing social awareness (e.g., the MeToo movement; Bongiorno et al., 2019), various studies indicate a subjective perception of SH, affected by variables such as cultural background, characteristics of the harasser and harassed, and characteristics of the evaluator (“bystander”), as well as other factors (Ohse and Stockdale, 2008; Angelone et al., 2009; Madan and Nalla, 2015). For instance, holding strict sex-role stereotypes was found to be related to higher SH tolerance (Ford and Donis, 1996; Russell and Trigg, 2004) and to perceiving different behaviors as less serious and harmful to victims (McCabe and Hardman, 2005; Shechory and Ben Shaul, 2013).

This is even more evident in “vague” behavioral situations open to multiple interpretations: for instance, sexist comments and jokes, applying pressure to go on a date, and sexual proposals (Osman, 2007; Ohse and Stockdale, 2008). While these behaviors may be perceived by men as flattering, flirtatious, and sexually attractive, women may perceive them as intimidating and harassing (Hands and Sanchez, 2000; Khoo and Senn, 2004).

Most research on SH has focused on female victims and male perpetrators, tapping into deep-rooted perceptions of gender-based aggression and victimhood (Bongiorno et al., 2019). Nonetheless, growing evidence suggests that men too are subjected to sexual abuse (Turchik and Edwards, 2012; Javaid, 2018). This has led to relatively limited research on social perceptions toward male victims (e.g., Shechory and Ben Shaul, 2013). Some studies found that when the perpetrator was portrayed as a man and the victim as a woman, both women and men were more inclined to perceive the behavior as SH (as compared to female perpetrator/male victim vignettes; Runtz and O’Donnell, 2003; McCabe and Hardman, 2005). In contrast, others found that this perception is only specific to men, while women perceived all SH gender configuration options as equally harassing and more serious than did men (Hendrix et al., 1998), including male victim cases (Shechory and Ben Shaul, 2013). Yet others showed that the more serious the behavior, the more it was perceived as SH, unrelated to victim or perpetrator gender (Wayne et al., 2001).

Just world theory (Lerner, 1980) may account for this subjective perception. It suggests that people’s behavior (e.g., blaming victims) is influenced by a need to feel that the world is a safe place, one governed by lawful moral outcomes – reward for good actions and punishment for evil actions. The need to belief in a just world (BJW) is a powerful motivator of behavior. Thus, if people are faced with salient threats to their BJW, they need to defend these beliefs and relieve emotional distress due to the attack against them. According to Lerner (1980, 1998), defensive behavior will take the form of one or more of three possible responses: prevention/restitution, avoidance, and victim derogation (blaming).

Empirical research provides evidence of the just world hypothesis (JWH) in varied situations related to rape and sexual abuse. When individuals are faced with a threat to their BJW (i.e., no retribution for a crime), they are more likely to assert that the victim is to blame (e.g., Hayes et al., 2013; Landström et al., 2016; Toews et al., 2019). For example, Strömwall et al. (2013) investigated the effects of BJW on blame attributions toward rape victims. They found that more blame was attributed to male victims, particularly by participants scoring high on BJW, and that participants high on BJW attributed more blame to the victim and less to the perpetrator.

In this study, we expand the relatively small body of research on perception of SH by legal experts, focusing on lawyers’ perceptions of SH victims and perpetrators, as well as their blame attributions toward them. We also examine whether perceptions and attributions are related to jurist BJW. Jurist perceptions and attributions, as well as BJW ratings, were compared to those of undergraduate students.

Examining the issue among professionals in law and its enforcement will help raise awareness of potential biases that might lead to miscarriage of justice. The present study attempted to address this by focusing on judgmental biases among lawyers. Specifically, lawyers and a comparison group of students were asked to judge male/female SH behavior toward a male/female victim. The vignette presented to the participants depicted indecent assault, which constitutes a violation of section 3.A2 (“indecent assault”) of the Sexual Harassment Prevention Act (1988). A number of hypotheses were tested. Based on the literature reviewed above, we expected that, in general, the victim’s gender would have an effect, such that there would be a greater tendency to perceive the behavior as SH when the victim is a woman and the perpetrator a man (as opposed to a male victim and female perpetrator). Nevertheless, the research premise is that lawyers, more than undergraduate students, will make more objective judgments.

The relatively little knowledge that exists on law enforcement professionals (mostly police officers) indicates judgmental biases associated with various variables, including stereotypical perceptions of rape. Thus, for example, there is evidence that police officer education and experience may be related to more objective judgments. Specifically, college education was related to lower levels of rape myth acceptance, higher professionalism and ethics, and improved work related behavior (Truxillo et al., 1998; Roberg and Bonn, 2004; Page, 2008). Similarly, police officers with experience in dealing with rape cases showed lower adherence to rape myths in comparison to inexperienced police officers (Page, 2007).

These findings suggest that knowledge and experience among police officers may be related to lower adherence to stereotypes and more objective work-related behavior. Thus, it is reasonable to assume that a possible corollary may exist among other legal professionals. Specifically, that lawyers’ knowledge and experience will be related to more objective judgments, compared with a comparison group of students with no legal knowledge and experience. The legal education and experience may make lawyers more aware of the possibility of women’s involvement in crime on the one hand and of male’s victimization on the other. This awareness may moderate bias related to the perpetrator/victim’s gender. Lawyers may also be more aware of unjustified accusations aimed at victims of SH. Thus, it was hypothesized that lawyers would exhibit more objective judgments with regard to both offender and victim blaming.

In addition, based on the literature on forensic judgments and BJW, it was further hypothesized that lawyers would exhibit stronger BJW than undergraduate students. To the best of our knowledge, the JWH has not been applied to lawyers. However, it is reasonable to assume that those who choose to be part of the justice system are motivated by the pursuit of justice. Therefore, it is quite possible that lawyers possess strong BJW. Finally, it was hypothesized that a positive correlation would be found between BJW and attributing blame to the victim.

## Method

### Participants

The study consisted of 211 participants: 91 lawyers, 39 (42.9%) women and 52 (57.1%) men, and 120 undergraduate students, 62 (51.7%) men and 58 (48.3%) women, undifferentiated by respondent sex (*Z* = 1.27, *p* > 0.05). Most of the lawyers and students were native born (94.5 and 95%, respectively). The mean age of the lawyers (*M* = 42.91, *SD* = 10.02) was significantly higher than that of the students [*M* = 24.12, *SD* = 2.51; *t*(98.59) = 17.49, *p* < 0.001]. A significant difference was found in the marital status of the two groups (*Z* = 8.59, *p* < 0.001). The rate of those married among the lawyers was significantly higher than among the students (67 vs. 14.2%, respectively). Furthermore, while 68.1% of the lawyers had children, only 5.8% of the students did (*Z* = 9.55, *p* < 0.001). All the students were studying for a bachelor’s degree in the social sciences at various schools. The lawyers had been working in their profession for a mean of 12.88 years (*SD* = 8.01).

### Instruments

The questionnaire booklet contained two vignettes, perceptions of the event as reflected by ratings of victim blame, ratings of perpetrator blame, an item on which the participants indicated their perception of the event described in the vignette as SH, the BJW questionnaire, and demographic data (e.g., gender, age, family status, education, and occupation) always in the same order.

#### Vignettes

In two different vignettes, the gender of the victim and of the perpetrator was manipulated. The victim was described as either male (David) or female (Sara). All other aspects were held constant. The vignettes depicted behavior that constitutes a violation of section 3.A2 (“indecent assault”) of the Sexual Harassment Prevention Act (1988). The following is a description of the vignette in which the victim was a woman and the perpetrator a man: “Sara was invited to a party at her friends’ house. One of the friends introduced Sara to David. The atmosphere was cheerful. Everyone danced, drank, and had a good time. Sara and David spent the evening together, talking flirtatiously, laughing, and touching each other. At a certain point, David told Sara that he needed some rest. He went into the bedroom. Sara followed him and asked if everything is okay. He said that he has a headache and asked if she could make him a cup of tea. Sara was glad to oblige. When she returned to the room she found him lying on the bed naked. She gave him the cup of tea and turned to leave the room. He pulled her to him, hugged her, and kissed her on the neck and tried to insert his hand into her pants. She told him that she was tired and wanted to go home. She freed herself from his embrace, fled the room, and left the party in an agitated state.”

Three scales were developed for use in the current study, based on the conceptualization of their contents.

#### Victim Blame Scale

The scale consisted of three items measuring aspects of victim blame: To what extent – Do you think that Sara (the victim) can be blamed for the event? Do you think that Sara could have prevented the event? Do you think that Sara provoked David’s (the perpetrator) behavior? Cronbach’s *α* was 0.72.

#### Perpetrator Blame Scale

The scale consisted of three items measuring aspects of perpetrator blame: To what extent – Do you think that the perpetrator can be blamed for the event? Do you think that Sara (the victim) should report the crime to the police? Do you think that David (the perpetrator) should be criminally prosecuted? Cronbach’s *α* was 0.78.

#### Judging the Behavior as SH

One item in which the participants indicated their perception of the event described in the vignette as SH under the criminal law.

Each of the items was rated on a five-point Likert-type scale ranging from 1 (*strongly disagree*) to 5 (*strongly agree*). Scores were comprised of item means, such that higher scores represented greater blame and greater belief that the behavior constitutes SH.

#### Beliefs in a Just World

The scale is a seven-item measure of the global beliefs in a just world scale (GBJWS; Lipkus, 1991). Responses are given on a seven-point Likert-type scale ranging from 1 (*strongly disagree*) to 7 (*strongly agree*). Higher scores indicate greater endorsement of BJW. Cronbach’s *α* was 0.84.

### Procedure

Data collection among the lawyers was goal-oriented, with only lawyers working in law and in the courts included in the study. Data collection among the undergraduate students was done by research assistants who met with students at their schools. Participation in the study was on a voluntary basis. All the respondents signed an informed consent form. All respondents were told that they could stop their participation in the study at any point, with no penalty. They were also promised that the data would be used for research purposes only.

The participants were assigned at random to one of the two questionnaire booklets. In the group of lawyers, 46 were presented with the woman as victim and 45 with the man as victim. In the group of students, 60 were presented with the woman as victim and 60 with the man as victim. No demographic differences were found within each group (lawyers/students) by victim gender in the vignette presented. The research design was approved by the university’s institutional ethics committee.

### Data Analysis

Data were analyzed with SPSS ver. 25. Internal consistencies were calculated, and variables were defined as item means. Variables of perception of the event were mathematically transformed due to non-normal distributions (Perpetrator blame and judging the behavior as SH were negatively skewed and were thus exponentially transformed. Victim blame was positively skewed and was thus log transformed). Differences in the perception of the event by group (lawyers/students) and the victim’s gender and their interaction were analyzed with analyses of covariance, controlling for the respondent’s gender (1-male and 0-female). Differences in BJW, by group and the victim’s gender, were analyzed with analyses of covariance, controlling for the respondent’s gender. The respondent’s gender was controlled for, rather than serving as another independent variable, to avoid cells that were too small. In addition, four multiple hierarchical regressions were calculated to assess the relationship between the perception of the event and BJW, group, victim’s gender, and their interactions. Power analysis was conducted with G*Power 3 (Faul et al., 2007). For analyses of covariance with main effects and interactions, low to medium effect size *f* = 0.20 (equals to *η*^2^ = 0.038), *α* = 0.05, and power = 0.80, a sample size of 199 participants was required. For a regression analysis with eight predictors (four predictors in the second step), low to medium effect size *f*^2^ = 0.065 (equals to *R*^2^ = 0.06), *α* = 0.05, and power = 0.80, a sample size of 189 participants was required.

## Results

Descriptive results (Table 1) revealed that BJW was moderate, perpetrator blame and judging the behavior as SH were high, and victim blame was low. Intercorrelations among the study variables demonstrate that victim blame was higher among students than lawyers, and that perpetrator blame and judging the behavior as SH were higher for female than male victims. Perpetrator blame was positively related with judging the behavior as SH, and both were negatively related with victim blame.

**Table 1:** Means, standard deviations (SDs), and intercorrelations among the study variables (*N* = 211).

	*M* (*SD*)	2	3	4	5	6	7
1.Group (lawyers)	0.43 (0.50)	−0.01	−0.09	0.11	0.12	−0.19^**^	0.12
2.Victim’s gender (male)	0.50 (0.50)		0.03	0.11	−0.33^***^	0.04	−0.43^***^
3.Respondent’s gender (male)	0.48 (0.50)			0.13	0.01	0.15^*^	−0.12
4.Belief in a just world (1–5)	3.61 (1.05)				0.05	−0.05	−0.04
5.Perpetrator blame (1–5)	3.79 (1.01)					−0.18^**^	0.60^***^
6.Victim blame (1–5)	1.89 (0.86)						−0.19^**^
7.Judging the behavior as SH (1–5)	4.36 (1.03)						

* *p* < 0.05;

** *p* < 0.01;

*** *p* < 0.001.

### Perpetrator Blame, Victim Blame, and Judging the Behavior as SH

Differences in perception of the event, by group and the victim’s gender, were analyzed with analyses of covariance, controlling for the participant’s gender (1-male and 0-female).

As can be seen from Table 2, victim blame attribution was lower than perpetrator blame. However, perpetrator blame attribution was higher among lawyers than among students (*p* = 0.036) and regarding female victims (i.e., male perpetrators) than male victims (*p* < 0.001). The interaction between group and victim’s gender was significant (*p* = 0.004), such that blame attribution by students toward female perpetrators (i.e., male victims) was lower than in all other sub-groups. Victim blame attribution was higher among students than among lawyers (*p* = 0.008). No other differences were found.

**Table 2:** Means, SDs, and *F* values for perception of the event, by group and the victim’s gender (*N* = 211).

Group	Lawyers (*N* = 91)			Students (*N* = 120)			Total (*N* = 211)			Difference		
Victim’s gender	Male *M* (*SD*) (*n* = 45)	Female *M* (*SD*) (*n* = 46)	Total *M* (*SD*)	Male *M* (*SD*) (*n* = 60)	Female *M* (*SD*) (*n* = 60)	Total *M* (*SD*)	Male *M* (*SD*)	Female *M* (*SD*)	Total *M* (*SD*)	Group *F*(1, 206) (*η*^2^)	Victim’s gender *F*(1, 206) (*η*^2^)	Group x victim’s gender *F*(1, 206) (*η*^2^)
Perpetrator blame	3.86 (0.96)	4.05 (0.98)	3.96 (0.97)	3.13 (0.93)	4.17 (0.83)	3.65 (1.02)	3.45 (1.01)	4.12 (0.9)	3.78 (1.01)	4.44^*^ (0.021)	23.77^***^ (0.104)	8.61^**^ (0.040)
Victim blame	1.63 (0.66)	1.83 (1.03)	1.73 (0.87)	2.11 (0.85)	1.91 (0.80)	2.01 (0.83)	1.90 (0.81)	1.87 (0.90)	1.89 (0.86)	7.11^**^ (0.033)	0.09 (0.001)	2.01 (0.010)
Judging the behavior as SH	4.29 (0.84)	4.70 (0.94)	4.49 (0.91)	3.77 (1.25)	4.73 (0.66)	4.25 (1.11)	3.99 (1.12)	4.72 (0.79)	4.36 (1.03)	2.91 (0.014)	43.89^***^ (0.176)	1.12 (0.005)

* *p* < 0.05;

** *p* < 0.01;

*** *p* < 0.001.

When analyzing the responses concerning the degree to which participants judged the described event as SH, no differences were found between the groups, with a high total mean (4.36, on a scale of 1–5). However, the behavior was regarded as SH more frequently in the case of female victims than of male victims (*p* < 0.001). The interaction between group and victim gender was non-significant.

### Belief in a Just World

Differences in BJW, by group and the victim’s gender, were assessed by analyses of covariance, controlling for the respondent’s gender (1-male and 0-female).

BJW was found to be higher among lawyers than among students (*p* = 0.020) and regarding male victims than regarding female victims (*p* = 0.025). The interaction between group and victim’s gender was non-significant (Table 3).

**Table 3:** Means, SDs, and *F* values for belief in a just world (BJW), by group and the victim’s gender (*N* = 211).

Group	Lawyers (*N* = 91)			Students (*N* = 120)			Total (*N* = 211)			Difference		
Victim’s gender	Male *M* (*SD*) (*n* = 45)	Female *M* (*SD*) (*n* = 46)	Total *M* (*SD*)	Male *M* (*SD*) (*n* = 60)	Female *M* (*SD*) (*n* = 60)	Total *M* (*SD*)	Male *M* (*SD*)	Female *M* (*SD*)	Total *M* (*SD*)	Group *F*(1, 206) (*η*^2^)	Victim’s gender *F*(1, 206) (*η*^2^)	Group x victim’s gender *F*(1, 206) (*η*^2^)
Belief in a just world	4.03 (1.08)	3.55 (1.24)	3.78 (1.18)	3.56 (0.85)	3.39 (0.92)	3.48 (0.88)	3.76 (0.98)	3.46 (1.07)	3.61 (1.03)	5.49^*^ (0.026)	5.11^*^ (0.025)	1.18 (0.006)

* *p* < 0.05.

### Relationship Between Belief in a Just World, Blame, and Judging Behavior as SH

Multiple regressions were calculated with the three variables of the perception of the event as dependent variables: Perpetrator blame, Victim blame, and Judging the behavior as SH. Independent variables were: BJW, group, victim’s gender, and the two-way and three-way interactions between BJW, group, and the victim’s gender. Results showed that BJW and its interactions with group and the victim’s gender were non-significant regarding all four dependent variables (Perpetrator blame: ΔAdj. *R*^2^ = −0.003, Δ*F*(4, 202) = 0.81, *p* = 0.521; Victim blame: ΔAdj. *R*^2^ = 0.010, Δ*F*(4, 202) = 1.58, *p* = 0.180; and Judging the behavior as SH: ΔAdj. *R*^2^ = −0.007, Δ*F*(4, 202) = 0.56, *p* = 0.694). Derived from the regression analysis, the findings showed that there is no relation between BJW and blame attributions, as well as between BJW and perceiving the behavior as SH.

## Discussion

The current research examined lawyers’ perceptions of SH, as well as their perceptions compared with those of students. The findings showed that both lawyers and students agreed that the described event should be viewed as SH. Nonetheless, while lawyers attributed more blame to perpetrators, students attributed more blame to victims, a finding that may indicate a more objective perception by lawyers versus students.

However, further thorough examination of the findings shows that the picture is not that clear. The interaction between the victim/offender’s gender and judging the behavior as SH, as well as examination of blame attributions, offers some interesting insights into how gender may affect lawyers’ perceptions. Similar to students, lawyers were more inclined to regard the behavior as SH when the vignette description depicted the perpetrator as a man (i.e., female victim) than as a woman (male victim).

Gender bias was also evident in the examination of blame attributions. It is not surprising that victim blame attribution was lower than perpetrator blame. Thus, also the finding whereby perpetrator blame attribution was higher among lawyers than among students. Nonetheless, in this case as well the findings indicate gender bias among the lawyers (although less than among the students) manifested in higher blame attributions when the victim was a woman and the perpetrator was a man.

It appears that the sociocultural model proposed in the 1980s by Tangri et al. (1982) is still relevant for explaining the gender differences found. According to the model, since women are taught and rewarded for passive and avoidant behavior and men are taught and rewarded for aggressive and dominating behavior, an interaction consisting of a woman sexually harassing a man contradicts the social conditioning and gender role instilled in us in the process of socialization. This social perception, which contradicts the situation described (where the woman is the aggressor), minimizes interpretation of the behavior portrayed as SH. The results may also be indicative of the participants’ stereotypes about the “typical” sexual offense victim, namely, that women, and not men, are the victims of such crimes. Drawing from rape research, men are not seen as “real” victims of rape, due to several myths and misconceptions, among which are that men always can defend themselves from an attack and want sex (Sleath and Bull, 2010).

System justification theory (SJT; Jost et al., 2004) posits that “people are motivated to justify and rationalize the way things are, so that existing social, economic, and political arrangements tend to be perceived as fair and legitimate” (Jost and Hunyady, 2005, p. 260). According to SJT, different ideologies, for example, opposition to equality, serve system justifying functions and are used to protect the *status quo* (Ståhl et al., 2010). Similarly, it may be argued that a woman sexually harassing a man contradicts and poses a threat to the traditional sexist conceptions of gender roles and behavior and to the stereotype that men cannot be victims of sexual offenses. Such a threat triggers attempts to justify the *status quo*, manifested in minimization of female aggressiveness and male victimization, as reflected in participant interpretation of the behavior as SH and their blame attributions.

The impact of the above mentioned stereotypes may be particularly true in Israel, a militarist society in which masculine norms of behavior, beginning with mandatory military service, are emphasized (Sarid, 2015; Shechory-Bitton and Jaeger, 2019). Although, in recent decades, Israeli women have gained more equality (gradually more women are occupying positions traditionally regarded as exclusively male; for example, in the army), the gender perception is still that women are weaker, more vulnerable, and therefore it is also harder to attribute to them behavior interpreted as SH. Gender inequality in Israeli society is evident in many Jewish families, as well as in the patriarchal Jewish religion. The Jewish family is tied to ancient religious traditions which are patriarchal in nature. The family is a dominant institution in Israeli society and in most Jewish Israeli families, religious ideology takes precedence over gender ideology. Religion in Israel is not separated from the state, and rabbinical courts have jurisdiction over matters of marriage and divorce. Their rulings represent Orthodox views of family and gender roles. All these central institutions (army, family, and religion) contribute to gender inequality and are the focus of feminist critiques (Lindsey, 2015).

Cultural characteristics related to gender stereotypes and sexist attitudes may influence perceptions of offenders and victim-blaming and may be particularly relevant to perceptions of sex offenses. It is generally assumed that attitudes toward rape and degrees of rape blame attribution vary on the basis of culture (Van der Bruggen and Grubb, 2014). This may also be the case for other sex offenses. It follows that offender and victim-blaming should be examined and interpreted in the context of examinees’ specific cultural background. Comparative studies are needed to account for culture-related differences (Van der Bruggen and Grubb, 2014).

The present research results are supported by previous findings in the research literature with general population samples. For example, a study that explored judgments of SH conducted in Israel (Shechory and Ben Shaul, 2013) found that, unrelated to their gender, respondents evaluated behaviors where the woman is the perpetrator as the least sexually harassing, particularly when the victim was a man. Similarly, others found that when the perpetrator was presented as a woman and the victim as a man, both women and men were less inclined to perceive the behavior as SH than when the perpetrator was presented as a man and the victim as a woman (Runtz and O’Donnell, 2003; McCabe and Hardman, 2005).

The resemblance described above between the responses of lawyers and students suggests that the social perception described exists in systems of law as well. Although the legal system emphasizes the neutrality and objectivity of its employees, and despite the conception that lawyers’ work has a rational basis (Bergman and Wettergren, 2015), lawyers may be biased – similar to anyone else (Knight et al., 2016).

This inference is further supported by lawyers’ perceptions regarding the needed legal measures. The questions on attributing blame to the perpetrator included, in addition to a direct question on evaluating the degree of blame, items concerning the justification for prosecuting the perpetrator for his/her behavior and whether the victim should report the crime to the police. The lawyers’ outlook, whereby a male perpetrator is more deserving to be prosecuted than a female perpetrator for the same act, is compatible with the judgment bias designated “chivalry bias” (Daly, 1987), whereby protective patriarchal outlooks related to gender stereotypes lead to judgment bias favoring female offenders within law enforcement and the justice system. Empirical studies support the idea that female offenders receive more lenient treatment than male offenders who carry out similar acts (Curry et al., 2004; Van Slyke and Bales, 2013; Shechory-Bitton and Zvi, 2019).

Notably, caution should be employed in interpreting the findings, as relatively few studies have explored perceptions of sex offenses by professionals in general and lawyers in particular. Most of them focused mainly on serious sex offenses of rape, female victims, and police officers (Sleath and Bull, 2017; Shechory-Bitton and Jaeger, 2019). Despite previous evidence that myths and stereotypes regarding sex offenses common in general society are also prevalent in the enforcement and legal systems (Rumney, 2009; Page, 2010; Shechory-Bitton and Jaeger, 2019), there is also evidence that this is true mainly in cases with no objective independent evidence [e.g., Closed-circuit TV (CCTV) or eyewitnesses]. This is what facilitates the impact of stereotypes and attitudes held by judges (Lovett and Kelly, 2009), jurors (Ellison and Munro, 2010), police officers (Hine and Murphy, 2019), and even offenders (Debowska et al., 2018b). Nevertheless, most sexual offenses are non-stranger assaults, and tangible evidence is therefore often lacking, thus making these cases at great risk for bias (Willmott et al., 2018). Yet, the more serious the offensive behavior described, the more limited the impact of the perpetrator and victim’s gender on the judgment passed. Gender differences were observed mainly in cases when the situation judged was vague and given to interpretation (for review, see, for example, O’Leary-Kelly et al., 2009).

While examining the relationship between BJW and victim blaming, we found that lawyers demonstrated higher levels of BJW than students. Still, our hypothesis concerning the correlation between BJW and perceiving the vignette as SH and blame attribution was not supported. Previous findings from studies that examined the relationship between BJW and victim blaming have been inconsistent in establishing whether just world theory provides a useful explanation for why rape victims are blamed (Sleath and Bull, 2010). Some research shows that stronger BJW was associated with blame attribution (see comprehensive review by Hafer and Bègue, 2005), as well as in cases of sexual assault (e.g., Strömwall et al., 2013; Landström et al., 2016; Adolfsson and Strömwall, 2017). However, in other studies, BJW did not predict victim or perpetrator blame and it was suggested that high BJW results in blaming only in certain circumstances, such as blaming victims of injustice, but not necessarily victims of rape (Ford et al., 1998; Sleath and Bull, 2010). Accordingly, using alternate BJW measures was suggested (Hafer and Sutton, 2016). For example, Maes’ (e.g., Maes, 1998; Maes and Schmitt, 1999) distinction between ultimate justice and imminent justice seems promising, as blaming victims and offenders are related to imminent rather than long-term justice (Hafer and Sutton, 2016). Yet others suggested that BJW may be more complex than allowed for by self-report questionnaires (Toews et al., 2019). In light of the criticism regarding the construct and measurement of the BJW, our research findings indicate the need for further research, using other measures of BJW.

The present study has several limitations. It was hard to recruit lawyers to participate in the study, and therefore, the sample size is relatively small. We also did not examine the lawyers’ field of specialty and activity. This is unfortunately reflective of how difficult it is to recruit meaningful sample sizes, especially on “sensitive” topics related to sexual assault (see also Sleath and Bull, 2012). Therefore, the findings and the ability to generalize from this sample to the entire population of lawyers must be treated with caution. In future research, differences within the population of lawyers should be examined as well, according to their specific field of occupation. It would be interesting to explore whether there is a difference between those engaged in criminal law versus civil law. Participants in the present study were both civil and criminal lawyers and all of them were asked to judge the case as a criminal case. Also examined should be the association with working with sexual offenders, judging sexual offenses, etc.

In addition, the comparison group consisted of undergraduate students. However, according to Van der Bruggen and Grubb (2014), observers from the general population make similar judgments of blame as do student participants. In addition, due to the sample size, it was not possible to examine gender differences between the respondents, although all the statistical analyses included controlling for the respondent’s gender. Various studies indicate gender differences with regard to judgment in situations involving sexual offenses (see, for example, Shaver, 1970; Grubb and Harrower, 2009; Vonderhaar and Carmody, 2015; Adolfsson and Strömwall, 2017).

Another limitation of the study is that the three scales – victim blame scale, perpetrator blame scale, and judging the behavior as SH – were developed for use in the current study, based on the conceptualization of their contents. Although they had reasonable internal consistency values, their items were not subjected to factor analysis. This is recommended for future studies. Another limitation concerns the issue of the power of the analyses. Sufficient power was found for the analyses of covariance, yet, as the regression analyses were found not significant, their power is low.

Finally, in future research, the topic should be examined in more diverse and larger populations. In the current study, no additional information was provided on the perpetrator or victim aside from that associated with the incident and their gender. There is a need for additional studies examining how professionals engaged in criminal law and lawyers, in particular, judge different situations of SH, as well as how various characteristics of the victim and offender affect these judgments. Among other things is the effect of homosexuality. The current study focused on harassment occurring in the context of heterosexual interaction. Future research should also look at the effect of same-sex harassment. Homophobia was found to strongly correlate with blaming homosexual male victims of rape (Van der Bruggen and Grubb, 2014), although examining perceptions of legal professional may yield different results.

In conclusion, although in recent decades there has been growing evidence, side by side with a rise in social awareness, that men too are sexually assaulted and harassed (Javaid, 2018; Weare, 2018), and, at the same time, that women are increasingly involved in crime, the current findings indicate that SH of a man by a woman is met with forgiveness, even by those in charge of maintaining justice. Women are still not perceived as perpetrators of SH but rather mainly as victims. These perceptions appear to reflect common gender stereotypes, are unfair toward men who are victims of sexual assault and do not advance the understanding and treatment of the sexual assault of men. Accordingly, it seems to be extremely important to expand educational and informational programs that will help change attitudes, undermine prevalent myths, and reduce the ability of both perpetrators and of society to devalue the victims. This is extremely important for male victims as this may release them from guilt feelings that arise due to social messages stemming from prejudice and stereotypical thinking. Such social change will assist both male and female victims, both of whom often avoid reporting the assault and seeking therapeutic help (for review, see Van der Bruggen and Grubb, 2014).

The findings concerning lawyers are partly encouraging (for example, attributing blame to the perpetrator rather than to the victims). However, they also demonstrate that some Israeli lawyers in the justice system exhibit stereotypical views capable of generating judgment bias and discriminatory treatment of SH by gender. Minimization of male sexual victimization may be particularly problematic within the legal system, as well as within other areas dealing with sexual offense victims (i.e., medicine and mental health). Reluctance to acknowledge that males can be victims of sex crimes might hinder the proper care that they should receive, as well as achieving justice (Romano and De Luca, 2001). Given the limited training provided to lawyers, expansion of educational systems in order to help change views and dispel common myths is of great significance. Further studies are needed in this area. Perceptions of legal professionals are rarely studied despite the clear importance of understanding the way they think about and feel toward victims and perpetrators, particularly in the context of sexual offenses, where tangible evidence is often lacking and stereotypes may exert great impact.

## Data Availability Statement

The datasets generated for this study are available on request to the corresponding author.

## Ethics Statement

The studies involving human participants were reviewed and approved by Ariel University. The patients/participants provided their written informed consent to participate in this study.

## Author Contributions

All authors listed have made a substantial, direct and intellectual contribution to the work, and approved it for publication.

### Conflict of Interest

The authors declare that the research was conducted in the absence of any commercial or financial relationships that could be construed as a potential conflict of interest.

## References

[ref1] Adolfsson K. Strömwall L. A. (2017). Situational variables or beliefs? A multifaceted approach to understanding blame attributions. Psychol. Crime Law 23, 527–552. 10.1080/1068316X.2017.1290236

[ref2] Angelone D. J. Mitchell D. Carola K. (2009). Tolerance of sexual harassment: a laboratory paradigm. Arch. Sex. Behav. 38, 949–962. 10.1007/s10508-008-9421-2, PMID: 19030980

[ref3] Bergman B. S. Wettergren Å. (2015). The emotional labour of gaining and maintaining access to the field. Qual. Res. 15, 688–704. 10.1177/1468794114561348

[ref4] Boduszek D. Debowska A. Jones A. D. Ma M. Smith D. Willmott D. (2019a). Prosocial video game as an intimate partner violence prevention tool among youth: a randomised controlled trial. Comput. Hum. Behav. 93, 260–266. 10.1016/j.chb.2018.12.028

[ref5] Boduszek D. Debowska A. Willmott D. Jones A. D. DeLisi M. Kirkman G. (2019b). Is female psychopathy linked with child abuse? An empirical investigation using a person-centered approach. J. Child Sex. Abus. 28, 708–725. 10.1080/10538712.2019.1592272, PMID: 30907696

[ref6] Bongiorno R. Langbroek C. Bain P. G. Ting M. Ryan M. K. (2019). Why women are blamed for being sexually harassed: the effects of empathy for female victims and male perpetrators. Psychol. Women Q. 44, 1–17. 10.1177/0361684319868730

[ref7] Craig J. M. Trulson C. R. DeLisi M. Caudill J. W. (2020). Toward an understanding of the impact of adverse childhood experiences on the recidivism of serious juvenile offenders. Am. J. Crim. Justice 1–16. 10.1007/s12103-020-09524-6

[ref8] Curry T. R. Lee G. Fernando Rodriguez S. (2004). Does victim gender increase sentence severity? Further explorations of gender dynamics and sentencing outcomes. Crime Delinq. 50, 319–343. 10.1177/0011128703256265

[ref9] Daly K. (1987). Discrimination in the criminal courts: family, gender, and the problem of equal treatment. Soc. Forces 66, 152–175. 10.2307/2578905

[ref10] Debowska A. Boduszek D. Sherretts N. Willmott D. Jones A. D. (2018a). Profiles and behavioral consequences of child abuse among adolescent girls and boys from Barbados and Grenada. Child Abus. Negl. 79, 245–258. 10.1016/j.chiabu.2018.02.018, PMID: 29486347

[ref11] Debowska A. Boduszek D. Willmott D. (2018b). Psychosocial correlates of attitudes toward male sexual violence in a sample of financial crime, property crime, general violent, and homicide offenders. Sex. Abus. 30, 705–727. 10.1177/1079063217691966, PMID: 29188756

[ref12] Dworkin E. R. Menon S. V. Bystrynski J. Allen N. E. (2017). Sexual assault victimization and psychopathology: a review and meta-analysis. Clin. Psychol. Rev. 56, 65–81. 10.1016/j.cpr.2017.06.002, PMID: 28689071 PMC5576571

[ref13] Ellison L. Munro V. E. (2010). A stranger in the bushes, or an elephant in the room? Critical reflections upon received rape myth wisdom in the context of a mock jury study. New Crim. Law Rev. 13, 781–801. 10.1525/nclr.2010.13.4.781

[ref14] Faul F. Erdfelder E. Lang A. -G. Buchner A. (2007). G*Power 3: a flexible statistical power analysis program for the social, behavioral, and biomedical sciences. Behav. Res. Methods 39, 175–191. 10.3758/BF03193146, PMID: 17695343

[ref15] Ford C. A. Donis F. J. (1996). The relationship between age and gender in workers’ attitudes toward sexual harassment. Aust. J. Psychol. 130, 627–633. 10.1080/00223980.1996.9915036, PMID: 9005253

[ref16] Ford T. M. Liwag-McLamb M. G. Foley L. A. (1998). Perceptions of rape based on sex and sexual orientation of the victim. J. Soc. Behav. Pers. 13, 253–263.

[ref17] Grubb A. R. Harrower J. (2009). Understanding attribution of blame in cases of rape: an analysis of participant gender, type of rape and perceived similarity to the victim. J. Sex. Aggress. 15, 63–81. 10.1080/13552600802641649

[ref18] Hafer C. L. Bègue L. (2005). Experimental research on just-world theory: problems, developments, and future challenges. Psychol. Bull. 131, 128–167. 10.1037/0033-2909.131.1.128, PMID: 15631556

[ref19] Hafer C. L. Sutton R. (2016). “Belief in a just world” in Handbook of social justice theory and research. eds. Sabbagh C. Schmitt M. (New York, NY: Springer), 145–160.

[ref20] Hammond L. Ioannou M. Fewster M. (2017). Perceptions of male rape and sexual assault in a male sample from the United Kingdom: barriers to reporting and the impacts of victimization. J. Investig. Psychol. Offender Profiling 14, 133–149. 10.1002/jip.1462

[ref21] Hands J. Z. Sanchez L. (2000). Badgering or bantering? Gender differences in experience of, and reactions to sexual harassment among U. S. high school students. Gend. Soc. 14, 718–746. 10.1177/089124300014006002

[ref22] Hayes R. M. Lorenz K. Bell K. A. (2013). Victim blaming others: rape myth acceptance and just world belief. Fem. Criminol. 8, 202–220. 10.1177/1557085113484788

[ref23] Hendrix W. H. Rueb J. D. Steel R. P. (1998). Sexual harassment and gender differences. J. Soc. Behav. Pers. 13, 235–252.

[ref24] Hine B. Murphy A. (2019). The influence of ‘High’ vs. ‘Low’ rape myth acceptance on police officers’ judgements of victim and perpetrator responsibility, and rape authenticity. J. Crim. Just. 60, 100–107. 10.1016/j.jcrimjus.2018.08.001

[ref25] Javaid A. (2018). The unheard victims: gender, policing and sexual violence. Polic. Soc. 30, 412–428. 10.1080/10439463.2018.1539484

[ref26] Jost J. T. Banaji M. R. Nosek B. A. (2004). A decade of system justification theory: accumulated evidence of conscious and unconscious bolstering of the status quo. Polit. Psychol. 25, 881–919. 10.1111/j.1467-9221.2004.00402.x

[ref27] Jost J. T. Hunyady O. (2005). Antecedents and consequences of system-justifying ideologies. Curr. Dir. Psychol. Sci. 14, 260–265. 10.1111/j.0963-7214.2005.00377.x

[ref28] Kassin S. M. Dror I. E. Kukucka J. (2013). The forensic confirmation bias: problems, perspectives, and proposed solutions. J. Appl. Res. Mem. Cogn. 2, 42–52. 10.1016/j.jarmac.2013.01.001

[ref29] Khoo P. N. Senn C. Y. (2004). Not wanted in the inbox!: evaluations of unsolicited and harassing e-mail. Psychol. Women 28, 204–214. 10.1111/j.1471-6402.2004.00137.x

[ref30] Knight C. Phillips J. Chapman T. (2016). Bringing the feelings back: returning emotions to criminal justice practice. Br. J. Commu. Justice 14, 45–58.

[ref31] Landström S. Strömwall L. A. Alfredsson H. (2016). Blame attributions in sexual crimes: effects of belief in a just world and victim behavior. Nord. Psychol. 68, 2–11. 10.1080/19012276.2015.1026921

[ref32] Lerner M. J. (1980). The belief in a just world: A fundamental delusion. New York: Plenum Press.

[ref33] Lerner M. J. (1998). “Two forms of belief in a just world: some thoughts on why and how people care about justice” in Responses to victimizations and belief in a just world. eds. Montada L. Lerner M. J. (New York: Plenum Press), 247–269.

[ref34] Lindsey L. L. (2015). Gender roles: A sociological perspective. New York, NY: Routledge.

[ref35] Lipkus I. (1991). The construction and preliminary validation of a global belief in a just world scale and the exploratory analysis of the multidimensional belief in a just world scale. Personal. Individ. Differ. 12, 1171–1178. 10.1016/0191-8869(91)90081-L

[ref36] Lovett J. Kelly L. (2009). Different systems, similar outcomes ? Tracking attrition in reported rape cases across Europe: Child & Woman Abuse Studies Unit, London Metropolitan University Available at: http://fellowship.birn.eu.com/en/file/show/jelena_blog4_lizkellyreport.pdf

[ref37] Madan M. Nalla M. K. (2015). Sexual harassment in public spaces: examining gender differences in perceived seriousness and victimization. Int. Crim. Justice Rev. 26, 80–97. 10.1177/1057567716639093

[ref81] Maes J. (1998). “Immanent justice and ultimate justice” in Responses to victimizations and belief in a just world. eds. Montada L. Lerner M. J. (New York, NY: Plenum Press), 9–40.

[ref82] Maes J. Schmitt M. (1999). More on ultimate and immanent justice: results from the research project “Justice as a problem within reunified Germany.” Soc. Justice Res. 12, 65–78. 10.1023/A:1022039624976, PMID: 16334516

[ref38] Magaji A. B. Ikhide J. E. Timur A. T. Timur S. (2019). “Sexual harassment in higher education: students’ perceptions and attitudes” in Global joint conference on industrial engineering and its application areas. eds. Calisir F. Korhan O. (Cham: Springer), 40–50.

[ref39] McCabe M. P. Hardman L. (2005). Attitudes and perceptions of workers to sexual harassment. J. Soc. Psychol. 145, 719–740. 10.3200/SOCP.145.6.719-740, PMID: 16334516

[ref40] Ohse D. M. Stockdale M. S. (2008). Age comparisons in workplace sexual harassment perceptions. Sex Roles 59, 240–253. 10.1007/s11199-008-9438-y

[ref41] O’Leary-Kelly A. M. Bowes-Sperry L. Bates C. A. Lean E. R. (2009). Sexual harassment at work: a decade (plus) of progress. J. Manag. 35, 503–536. 10.1177/0149206308330555

[ref42] Osman S. L. (2007). The continuation of perpetrator behaviors that influence perceptions of sexual harassment. Sex Roles 56, 63–69. 10.1007/s11199-006-9149-1

[ref43] Page A. D. (2007). Behind the blue line: investigating police officers; attitudes toward rape. J. Polic. Commun. Psychol. 22, 22–32. 10.1007/s11896-007-9002-7

[ref44] Page A. D. (2008). Judging women and defining crime: police officers’ attitudes toward women and rape. Sociol. Spectr. 28, 389–411. 10.1080/02732170802053621

[ref45] Page A. D. (2010). True colors: police officers and rape myth acceptance. Fem. Criminol. 5, 315–334. 10.1177/1557085110384108

[ref46] Peer E. Gamliel E. (2013). Heuristics and biases in judicial decisions. Court Rev. 49, 114–118.

[ref47] Perilloux C. Duntley J. D. Buss D. M. (2014). Blame attribution in sexual victimization. Personal. Individ. Differ. 63, 81–86. 10.1016/j.paid.2014.01.058

[ref48] Prevention of Sexual Harassment Law 5758 (1998). Available at: https://www.ilo.org/dyn/natlex/natlex4.detail?p_isn=57358&p_lang=en (Accessed June 26, 2020).

[ref49] Roberg R. Bonn S. (2004). Higher education and policing: where are we now? Policing 27, 469–486. 10.1108/13639510410566226

[ref50] Romano E. De Luca R. (2001). Male sexual abuse: a review of effects, abuse characteristics, and links with later psychological functioning. Aggress. Violent Behav. 6, 55–78. 10.1016/S1359-1789(99)00011-7

[ref51] Rumney P. N. S. (2009). Gaymale rape victims: law enforcement, social attitudes and barriers to recognition. Int. J. Hum. Rights 13, 233–250. 10.1080/13642980902758135

[ref52] Runtz M. J. O’Donnell C. W. (2003). Students’ perceptions of sexual harassment: is it harassment only if the offender is a man and the victim is a woman? J. Appl. Soc. Psychol. 33, 963–982. 10.1111/j.1559-1816.2003.tb01934.x

[ref53] Russell B. L. Trigg K. Y. (2004). Tolerance of sexual harassment: an examination of gender differences, ambivalent sexism, social dominance, and gender roles. Sex Roles 50, 565–573. 10.1023/B:SERS.0000023075.32252.fd

[ref54] Sarid O. (2015). Assessment of anger terms in Hebrew: a gender comparison. Aust. J. Psychol. 149, 303–324. 10.1080/00223980.2013.877867, PMID: 25590344

[ref85] Sexual Harassment Prevention Act (1988). Sexual Harassment Prevention *Law*. 5758–1998. SH 166.

[ref55] Shaver K. G. (1970). Defensive attribution: effects of severity and relevance on the responsibility assigned for an accident. J. Pers. Soc. Psychol. 14, 101–113. 10.1037/h0028777

[ref56] Shaw J. Campbell R. Cain D. Feeney H. (2017). Beyond surveys and scales: how rape myths manifest in sexual assault police records. Psychol. Violence 7, 602–614. 10.1037/vio0000072

[ref57] Shechory M. Ben Shaul D. (2013). Perceptions and attitudes to sexual harassment: an examination of gender differences and the gender composition of the harasser-target dyad. J. Appl. Soc. Psychol. 43, 2136–2145. 10.1111/jasp.12166

[ref58] Shechory-Bitton M. Jaeger L. (2019). “It can’t be rape”: Female vs. male rape myths among Israeli police officers. J. Polic. Crim. Psychol. 10.1007/s11896-019-09327-4 [Epub ahead of print]

[ref59] Shechory-Bitton M. Zvi L. (2015). The effect of offender’s attractiveness and subject’s gender on judgments in swindling. Psychiatry Psychol. Law 22, 559–570. 10.1080/13218719.2014.960037

[ref60] Shechory-Bitton M. Zvi L. (2016). Does offenders’ facial attractiveness affect police officers’ judgment? Psychiatry Psychol. Law 23, 588–601. 10.1080/13218719.2015.1084660

[ref61] Shechory-Bitton M. Zvi L. (2019). Chivalry and attractiveness bias in police officer forensic judgments in Israel. J. Soc. Psychol. 5, 503–517. 10.1080/00224545.2018.1509043, PMID: 30152730

[ref62] Sleath E. Bull R. (2010). Male rape victim and perpetrator blaming. J. Interpers. Violence 25, 969–988. 10.1177/0886260509340534, PMID: 19738198

[ref63] Sleath E. Bull R. (2012). Comparing rape victim and perpetrator blaming in a police officer sample: differences between police officers with and without special training. Crim. Justice Behav. 39, 646–665. 10.1177/0093854811434696

[ref64] Sleath E. Bull R. (2015). A brief report on rape myth acceptance: differences between police officers, law students, and psychology students in the United Kingdom. Violence Vict. 30, 136–147. 10.1891/0886-6708.VV-D-13-00035, PMID: 25774419

[ref65] Sleath E. Bull R. (2017). Police perceptions of rape victims and the impact on case decision making: a systematic review. Aggress. Violent Behav. 34, 102–112. 10.1016/j.avb.2017.02.003

[ref66] Ståhl T. Eek D. Kazemi A. (2010). Rape victim blaming as system justification: the role of gender and activation of complementary stereotypes. Soc. Justice Res. 23, 239–258. 10.1007/s11211-010-0117-0

[ref67] Strömwall L. A. Alfredsson H. Landström S. (2013). Rape victim and perpetrator blame and the just world hypothesis: the influence of victim gender and age. J. Sex. Aggress. 19, 207–217. 10.1080/13552600.2012.683455

[ref68] Suma S. Sandhya N. Vevek N. (2017). A comparative study to analyse the effectiveness of sexual harassment policies of IT and non IT companies. Ushus J. Bus. Manag. 16, 1–11. 10.12725/ujbm.39.1

[ref69] Tangri S. S. Burt M. R. Johnson L. B. (1982). Sexual harassment at work: three explanatory models. J. Soc. Issues 38, 33–54. 10.1111/j.1540-4560.1982.tb01909.x

[ref70] Temkin J. Krahe B. (2007). Sexual assault and the justice gap: A question of attitude. Oxford: Hart.

[ref71] Toews K. Cummings J. A. Zagrodney J. L. (2019). Mother blame and the just world theory in child sexual abuse cases. J. Interpers. Violence 34, 4661–4686. 10.1177/0886260516675922, PMID: 27821642

[ref83] Truxillo D. M. Bennett S. R. Collins M. L. (1998). College education and police job performance: a ten year study. Public Pers. Manag. 27, 269–280. 10.1177/009102609802700211, PMID: 27821642

[ref72] Turchik J. A. Edwards K. M. (2012). Myths about male rape: a literature review. Psychol. Men Masculinity 13, 211–226. 10.1037/a0023207

[ref73] Van der Bruggen M. Grubb A. R. (2014). A review of the literature relating to rape victim blaming: an analysis of the impact of observer and victim characteristics on attribution of blame in rape cases. Aggress. Violent Behav. 19, 523–531. 10.1016/j.avb.2014.07.008

[ref74] Van Slyke S. R. Bales W. D. (2013). Gender dynamics in the sentencing of white-collar offenders. Crim. Justice Stud. 26, 168–196. 10.1080/1478601X.2012.729707

[ref75] Venema R. M. (2016). Making judgments: how blame mediates the influence of rape myth acceptance in police response to sexual assault. J. Interpers. Violence 34, 2697–2722. 10.1177/0886260516662437, PMID: 27495113

[ref76] Vonderhaar R. L. Carmody D. C. (2015). There are no “innocent victims”: the influence of just world beliefs and prior victimization on rape myth acceptance. J. Interpers. Violence 30, 1615–1632. 10.1177/0886260514549196, PMID: 25236676

[ref77] Wayne J. H. Riordan C. M. Thomas K. M. (2001). Is all sexual harassment viewed the same? Mock juror decisions in same‐ and cross-gender cases. J. Appl. Psychol. 86, 179–187. 10.1037/0021-9010.86.2.179, PMID: 11393431

[ref78] Weare S. (2018). ‘Oh you’re a guy, how could you be raped by a woman, that makes no sense’: towards a case for legally recognising and labelling ‘forced-to-penetrate’cases as rape. Int. J. Law Context 14, 110–131. 10.1017/S1744552317000179

[ref79] Willmott D. Boduszek D. Debowska A. Woodfield R. (2018). Introduction and validation of the juror decision scale (JDS): an empirical investigation of the story model. J. Crim. Just. 57, 26–34. 10.1016/j.jcrimjus.2018.03.004

[ref80] Willness C. R. Steel P. Lee K. (2007). A meta-analysis of the antecedents and consequences of workplace sexual harassment. Pers. Psychol. 60, 127–162. 10.1111/j.1744-6570.2007.00067.x

